# Zac1 regulates IL-11 expression in osteoarthritis

**DOI:** 10.18632/oncotarget.25980

**Published:** 2018-08-21

**Authors:** Chun-Lin Kuo, Shu-Ting Liu, Yung-Lung Chang, Chia-Chun Wu, Shih-Ming Huang

**Affiliations:** ^1^ Graduate Institute of Medical Sciences, National Defense Medical Center, Taiwan, Republic of China; ^2^ Department of Orthopaedic Surgery, Tri-Service General Hospital, National Defense Medical Center, Taiwan, Republic of China; ^3^ Department of Biochemistry, National Defense Medical Center, Taiwan, Republic of China

**Keywords:** zac1, IL-11, HeLa, osteoarthritis, IL-6

## Abstract

Interleukin (IL)-11, a member of the IL-6 family of cytokines, exerts pleiotropic effects under normal and various disease conditions. We assessed *IL-11* expression regulation and the IL-11/IL-6 ratio in osteoarthritis (OA) to better guide clinical therapeutic decision-making. Our findings suggest that Zac1, a zinc finger protein that regulates apoptosis and cell cycle arrest, is a transcription factor regulating *IL-11* expression. Zac1 overexpression or knockdown respectively induced or suppressed *IL-11* expression in HeLa cells. Zac1 acted synergistically with AP-1, human papillomavirus E2, and hypoxia inducible factor 1 alpha (HIF1α). *IL-11* expression under various conditions, including hypoxia or treatment with phorbol 12-myristate 13-acetate or copper sulfate. Recombinant IL-11-induced phosphorylation of signal transducer and activator of transcription 3 at tyrosine 705 was reduced in a dose-dependent manner in HeLa cells. Cross-talk between Zac1, IL-11, p53, and suppressor of cytokine signaling 3 was differentially affected by copper sulfate, digoxin, and caffeine. Finally, aggressive vs. conventional treatment of OA patients was primarily determined by IL-6 levels. However, we suggest that OA patients with higher IL-11 levels may respond well to conventional treatments, even in the presence of high IL-6.

## INTRODUCTION

Interleukin (IL)-11 is a member of the IL-6 family of cytokines and exerts pleiotropic effects, conferring protection in the intestine, promoting tumorigenesis in gastrointestinal, breast, and thyroid tumors, regulating macrophage differentiation, and stimulating hemopoiesis and thrombopoiesis; however, *IL-11* regulatory mechanisms *in vivo* remain unclear. While recent progress has been made toward elucidating the IL-6 and IL-11 modes of action [1–4], the effects of these molecules on certain human diseases, including osteoarthritis (OA), are not well understood. OA is characterized by joint pain and varying degrees of functional limitation in the peripheral joints, especially the knee [5], resulting from cartilage degradation and erosion [6–10].

IL-11 shares characteristics with both immune-regulatory (IL-6) and neuro-protective (leukemia inhibitory factor and ciliary neurotrophic factor) members of this cytokine family. IL-11 forms a hexameric signaling complex similar to that of IL-6, but the IL-11 receptor complex contains a single glycoprotein 130 (gp130) chain and a cytokine-specific receptor α chain. Janus kinase-signal transducer and activator of transcription (JAK-STAT) signaling, including dissociation of receptor-associated JAK molecules, endocytosis of the receptor complex, and nuclear export of activated STAT molecules. Suppressor of cytokine signaling 3 (SOCS3) limits gp130-mediated signaling in a negative-feedback loop by binding to a tyrosine residue at position 757 in mice and 759 in humans [11–13].

Zac1 (also known as pleomorphic adenoma gene-like 1, PLAGL1) is a zinc-finger protein that regulates apoptosis and cell cycle arrest 1. Zac1 and p53 were identified through induction of type I pituitary adenylate cyclase-activating polypeptide receptor (PACAP1-R) expression [14–16]. As a transcription factor, Zac1 appears to recognize GC-rich DNA elements within the *PACAP1-R*, *cytokeratin*, *peroxisome proliferator-activated receptor-γ* (*PPAR-γ*), *insulin-regulated glucose transporter GLUT4*, *atrial natriuretic factor* (*ANF*), and *SOCS3* genes [14, 17–21]. We previously found that the Zac1 N-terminal motif is important for dimerization, nuclear sub-cellular localization, and protein-protein interactions [20, 22–24]. Zac1 is also a transcription cofactor for p53, human papillomavirus (HPV) oncoproteins (E2, E6, and E7), nuclear receptors (NRs), and NR coactivators for AP-1, CBP, p300, PML, Sp1, and SUMO [24–32]. In some cases, Zac1 may act as a transcriptional repressor via recruitment of histone deacetylase 1 or the NF-κB [21, 33, 34].

*IL-11* transcription is largely dependent upon AP-1 transcription factors. Studies also demonstrate that CREB, SMADs, and NF-κB [35–38]. Several other transcription factors, including AP-1, NF-κB, and CCAAT/enhancer-binding protein β (C/EBPβ), also bind the *IL-6* promoter region [39]. Among these, NF-κB activation, particularly via Toll-like receptor 4, is considered the most important trigger for IL-6 transcription and secretion [40]. These findings suggest that Zac1 might be a transcription factor regulating *IL-11* expression.

IL-6 and IL-11 are largely absent from body fluids of healthy individuals [39, 41]. However, a wide variety of cell types produce these cytokines following an appropriate stimulus. In contrast to the plethora of cell types that can produce cytokines, expression of their respective receptors is much more restricted. This limits the spectrum of cells that can be directly activated by IL-11 and IL-6. Dysregulation of IL-6 and IL-11 signaling contributes to several diseases, such as inflammatory bowel disease, osteoporosis, rheumatoid arthritis, and various types of cancer [11, 12]. In particular, the relationship between IL-6 and IL-11 in human articular tissues remains unclear. This study assessed *IL-11* regulatory mechanisms and compared clinical IL-6 and IL-11 levels to better elucidate the value of the IL-6/IL-11 ratio in OA patients. Our findings provide novel insights into therapeutic strategies for treating IL-6-related disorders.

## RESULTS

### *IL-11* and *IL-11 receptor α* (*IL-11Rα*) were induced in Zac1 stably-expressing HeLa cells

We overexpressed Zac1 in the HPV type 18 infected cervical carcinoma cell line, HeLa, to identify its potential target genes. Our mRNA expression array revealed that 890 genes were upregulated and 385 were downregulated. Table 1 lists the top 20 up- and downregulated genes. *IL-11* and *IL-11Rα* were dramatically induced by Zac1 in HeLa cells (Table 2). We then addressed whether or not Zac1 directly targeted *IL-11*.

**Table 1:** Top 20 ranking up- and down-regulation by Zac1 in HeLa cell line

Ranking	log_2_ (Fold)	*P*-value (Differentially expressed)	Gene symbol	Description
1	6.21	1.34E-34	BIRC7	baculoviral IAP repeat-containing 7
2	5.62	1.29E-28	CALHM3	calcium homeostasis modulator 3
3	5.41	5.73E-30	ITIH3	inter-alpha (globulin) inhibitor H3
4	5.41	1.86E-29	HM13	histocompatibility (minor) 13
5	5.31	2.11E-34	COL9A3	collagen, type IX, alpha 3
6	5.21	5.54E-24	IL-11	interleukin 11
7	5.15	7.24E-26	LGALS7B	lectin, galactoside-binding, soluble, 7B
8	4.98	4.20E-23	BIK	BCL2-interacting killer (apoptosis-inducing)
9	4.91	7.75E-31	CLIC3	chloride intracellular channel 3
10	4.74	4.23E-20	AMBP	alpha-1-microglobulin/bikunin precursor
11	4.67	1.52E-22	S100A2	S100 calcium binding protein A2
12	4.66	1.70E-25	KREMEN1	kringle containing transmembrane protein 1
13	4.65	6.38E-24	GPR153	G protein-coupled receptor 153
14	4.59	7.94E-29	FAM69B	family with sequence similarity 69, member B
15	4.58	6.85E-14	TNNT3	troponin T type 3 (skeletal, fast)
16	4.53	5.17E-26	GPT	glutamic-pyruvate transaminase (alanine aminotransferase)
17	4.53	1.30E-12	LAT2	linker for activation of T cells family, member 2
18	4.45	8.43E-30	CRLF1	cytokine receptor-like factor 1
19	4.44	1.75E-25	C6orf218	chromosome 6 open reading frame 218
20	4.38	3.82E-22	KCNQ2	potassium voltage-gated channel, KQT-like subfamily, member 2
1	−3.66	1.31E-29	C8orf22	chromosome 8 open reading frame 22
2	−3.30	2.94E-19	AKR1C3	aldo-keto reductase family 1, member C3
3	−3.00	3.99E-19	RNF182	ring finger protein 182
4	−2.66	1.81E-10	TSPAN8	tetraspanin 8
5	−2.54	2.15E-22	METTL7A	methyltransferase like 7A
6	−2.35	8.04E-11	COL3A1	collagen, type III, alpha 1
7	−2.35	3.35E-17	FAM64A	family with sequence similarity 64, member A
8	−2.31	2.89E-14	DOCK11	dedicator of cytokinesis 11
9	−2.30	7.02E-13	TDRD3	tudor domain containing 3
10	−2.23	4.75E-13	MAP2K6	mitogen-activated protein kinase kinase 6
11	−2.15	5.55E-11	NOP56	NOP56 ribonucleoprotein homolog (yeast)
12	−2.13	9.21E-09	MMP28	matrix metallopeptidase 28
13	−2.03	4.02E-11	MAMLD1	mastermind-like domain containing 1
14	−1.99	6.99E-16	SLC25A14	solute carrier family 25, member 14
15	−1.97	4.97E-13	DTX4	deltex homolog 4 (Drosophila)
16	−1.96	2.20E-12	RRM2	ribonucleotide reductase M2
17	−1.96	6.14E-16	PRAME	preferentially expressed antigen in melanoma
18	−1.95	2.93E-14	SNAP25	synaptosomal-associated protein, 25kDa
19	−1.92	0.001141	PDGFRL	platelet-derived growth factor receptor-like
20	−1.92	3.43E-11	COX7B2	cytochrome c oxidase subunit VIIb2

**Table 2:** IL-11 and its receptor (IL-11Rα) differentially expressed in the Zac1-stable HeLa cells

Gene name	log_2_ (Fold)	*P*-value (Differentially expressed)
IL-11	5.21	5.5E-24
IL-11 receptor, alpha (IL-11Rα)	2.61	1.1E-15

**Figure 1:**
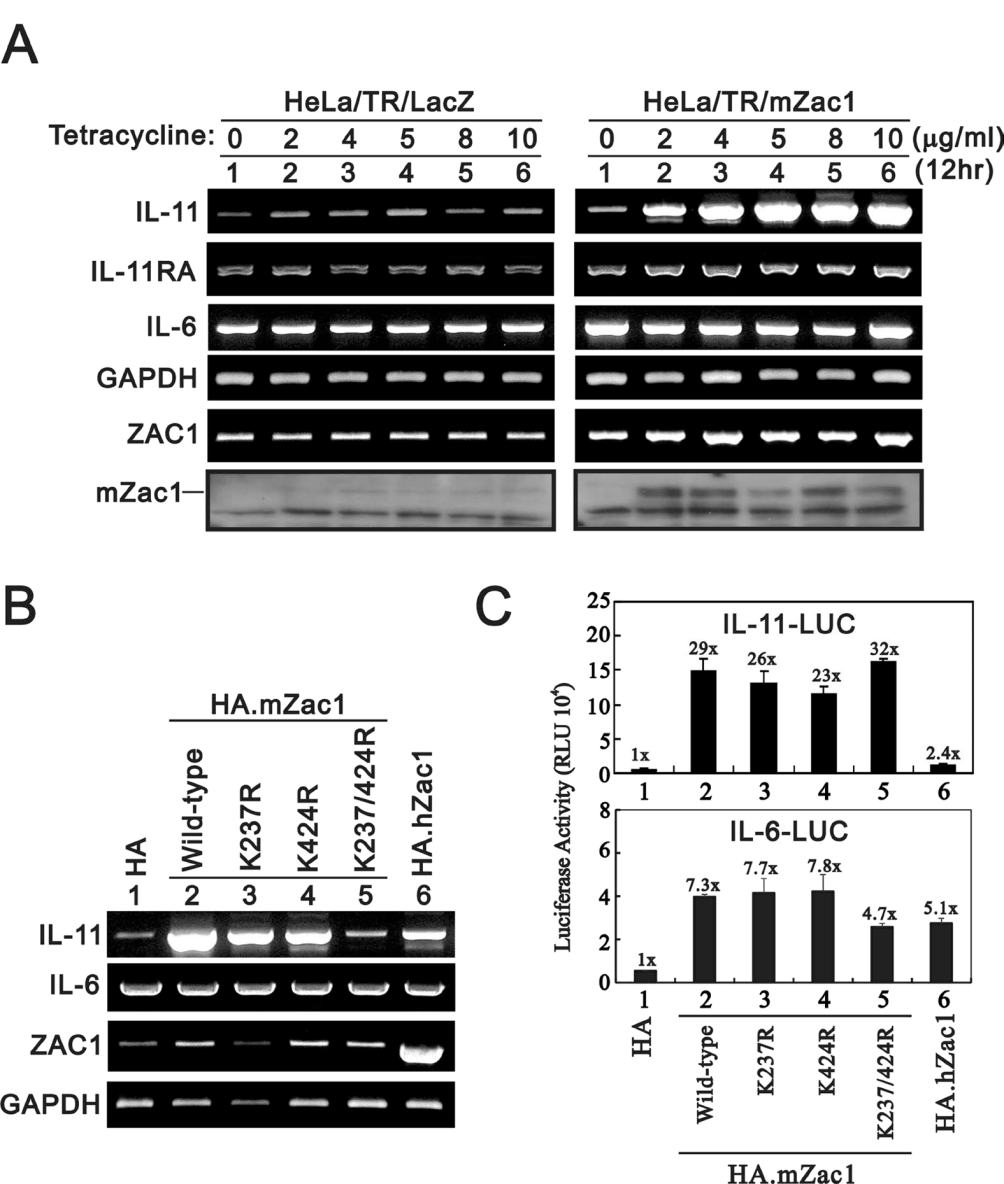
Effects of overexpressing Zac1 on the *IL-11* mRNA and promoter in HeLa cells (**A**) HeLa/TR/mZac1 or HeLa/TR-LacZ control cells were induced for 12 h using the indicated amounts of Dox. The cells were collected and subjected to RT-PCR analysis of *IL-11*, *IL-6*, *IL-11Rα, Zac1,* and *GAPDH* (loading control) mRNA expression, and to immunoblot analysis for detection of Zac1 protein. (**B**) HeLa cells were transiently transfected with 0.5 μg of the indicated pSG5.HA.Zac1 for 36 h. The cells were then collected and subjected to RT-PCR analysis of *IL-11*, *IL-6*, *IL-11Rα, Zac1, and GAPDH* (loading control) expression. (**C**) HeLa cells were transiently transfected with 0.5 μg of IL-11-LUC or IL-6-LUC along with 0.5 μg of the indicated pSG5.HA Zac1 for 36 h prior to use in luciferase reporter assays. The numbers above the columns indicate the luciferase activity relative to an index of 1 assigned to the reporter with empty vector. Results are representative of three independent experiments.

In the tetracycline-inducible *Zac1* expression system, we observed that *IL-11* expression was dependent on doxycycline (Dox) concentration (Figure 1A). *IL-6* was not induced under these experimental conditions. We previously showed that the two SUMO-binding lysine residues in Zac1, K237 and K424, repress Zac1 transactivation activity [32]. We examined the importance of these two sites with respect to IL-11 regulation. The Zac1 K237/K424A double mutant did not induce *IL-11* expression in HeLa cells (Figure 1B). K237A or K424A single mutants reduced Zac1-induced *IL-11* expression by half. RT-PCR data were consistent with the reduced Zac1 transactivation activity and no effect on *IL-6* expression.

We observed different responsive patterns for wild-type and mutant Zac1 in the *IL-11* and *IL-6* promoter reporter systems (Figure 1C). Double and single mutant Zac1 had similar effects as wild-type Zac1 on *IL-11* promoter activity, and all Zac1 constructs had differential transactivation activities on *IL-6* promoter activity in HeLa cells. The consistent enhancement on gene expressions by mouse Zac1 was higher than that of human Zac1 [14, 16].

**Figure 2:**
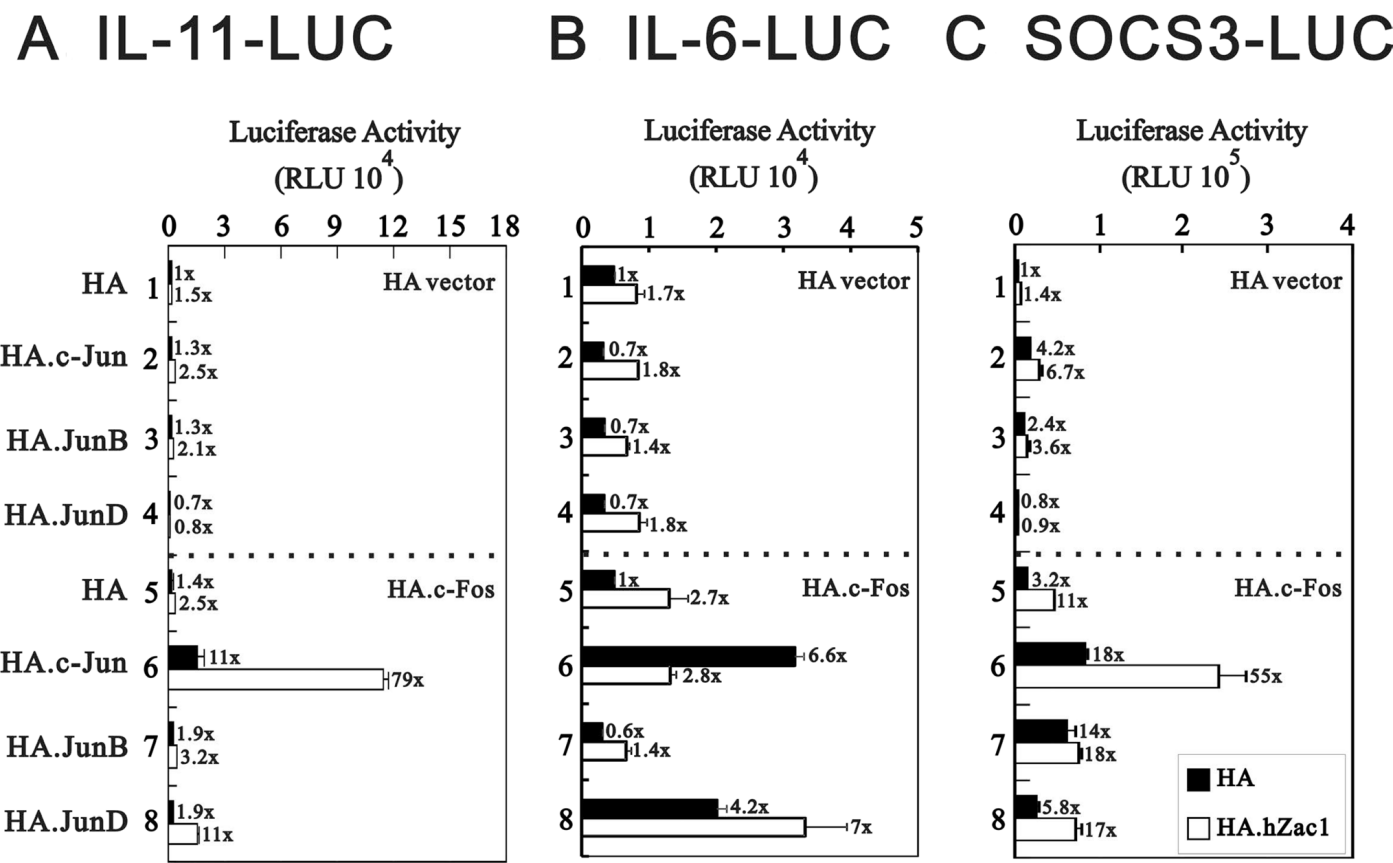
Effect of cross-talk between Zac1 and AP1 on the promoter activities of *IL-11*, *IL-6* and *SOCS3* HeLa cells were transiently transfected for 36 h with 0.5 μg of *IL-11*-LUC (**A**), *IL-6*-LUC (**B**), or *SOCS3*-LUC (**C**) along with 0.5 μg of pSG5.HA, pSG5.HA.c-Jun, pSG5.HA.JunB, and pSG5.HA.JunD in the absence and presence of 0.5 μg of pSG5.HA, pSG5.HA.c-Fos or pSG5.HA.hZac1. The transfectants were then harvested for luciferase reporter assays. The numbers above the columns (A–C) indicate the luciferase activity relative to an index of 1 assigned to the reporter with empty vector. Results are representative of three independent experiments.

In addition to regulating *IL-11*, AP-1 also binds the *IL-6* promoter region [39]. Our previous work revealed that Zac1 might regulate AP-1 function via enhancement of c-Jun and c-Fos transactivation activities [29]. Here, we transiently transfected various c-Jun family proteins, including c-Jun, JunB, and JunD, with c-Fos, Zac1, or both into HeLa cells to examine their effects on *IL-11*, *IL-6*, and *SOCS3* promoter activities. Our data revealed that Zac1 worked synergistically with c-Jun/c-Fos to enhance *IL-11* and *SOCS3* promoter activities (Figure 2A and 2C), whereas Zac1 suppressed the effects of c-Jun/c-Fos on *IL-6* promoter activity (Figure 2B). Zac1 selectively synergistically worked with JunD/c-Fos to enhance *IL-6* promoter activity (Figure 2B).

### Zac1 acted synergistically with HPV E2 to enhance *IL-11* promoter activity in HeLa cells

**Figure 3:**
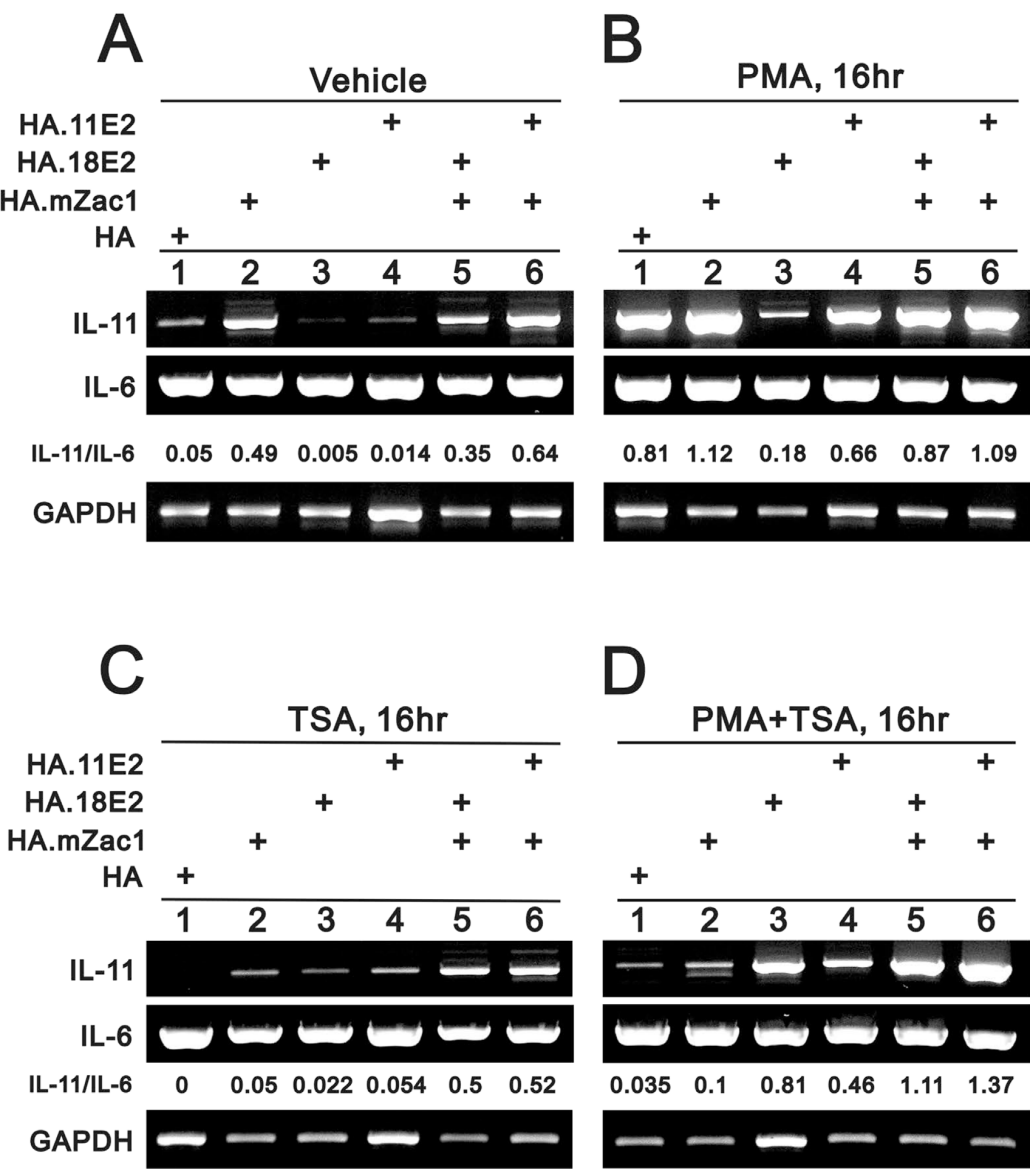
The rescue effect of Zac1 on HPV E2 proteins suppresses *IL-11* mRNA expression HeLa cells were transiently transfected for 20 h with 0.5 μg of pSG5.HA, pSG5.HA.mZac1, pSG5.HA.11E2, and pSG5.HA.18E2 and then treated with vehicle (**A**), PMA (**B**), TSA (**C**), or PMA (**D**) for 16 h. The cells were then collected and subjected to RT-PCR analysis of *IL-11*, *IL-6*, *and GAPDH* (loading control) expression. The IL-11/IL-6 ratio is presented as fold change. Results are representative of three independent experiments.

HeLa cells are an HPV-infected cervical cancer cell line containing integrated HPV 18 DNA [42]. The integration interrupts the E2 open reading frame and the encoded protein is not produced. We examined the functional role of HPV E2 in *IL-11* gene regulation. Our data showed that high risk HPV type 18 E2 (HPV18E2) and low risk HPV type 11 E2 (HPV11E2) proteins downregulated *IL-11* expression, and Zac1 could rescue this suppressive effect (Figure 3A). Phorbol 12-myristate 13-acetate (PMA) enhanced endogenous *IL-11* expression (Figure 3A and 3B, lane 1). The histone deacetylase inhibitor, trichostatin A (TSA), suppressed endogenous *IL-11* expression (Figure 3A and 3C, lane 1). TSA also suppressed Zac1-induced *IL-11* expression and Zac1 rescue of HPV E2 protein-induced *IL-11* suppression, but reversed the functional roles of HPV E2 proteins to induce, rather than repress, *IL-11* expression (Figure 3A and 3C, lanes 1, 3, 4). PMA and TSA combined selectively suppressed Zac1 and enhance HPV18 E2 functionality with respect to *IL-11* expression (Figure 3A and 3D, lanes 1, 3, 5). IL-6 expression was not affected by these treatments (Figure 3).

**Figure 4:**
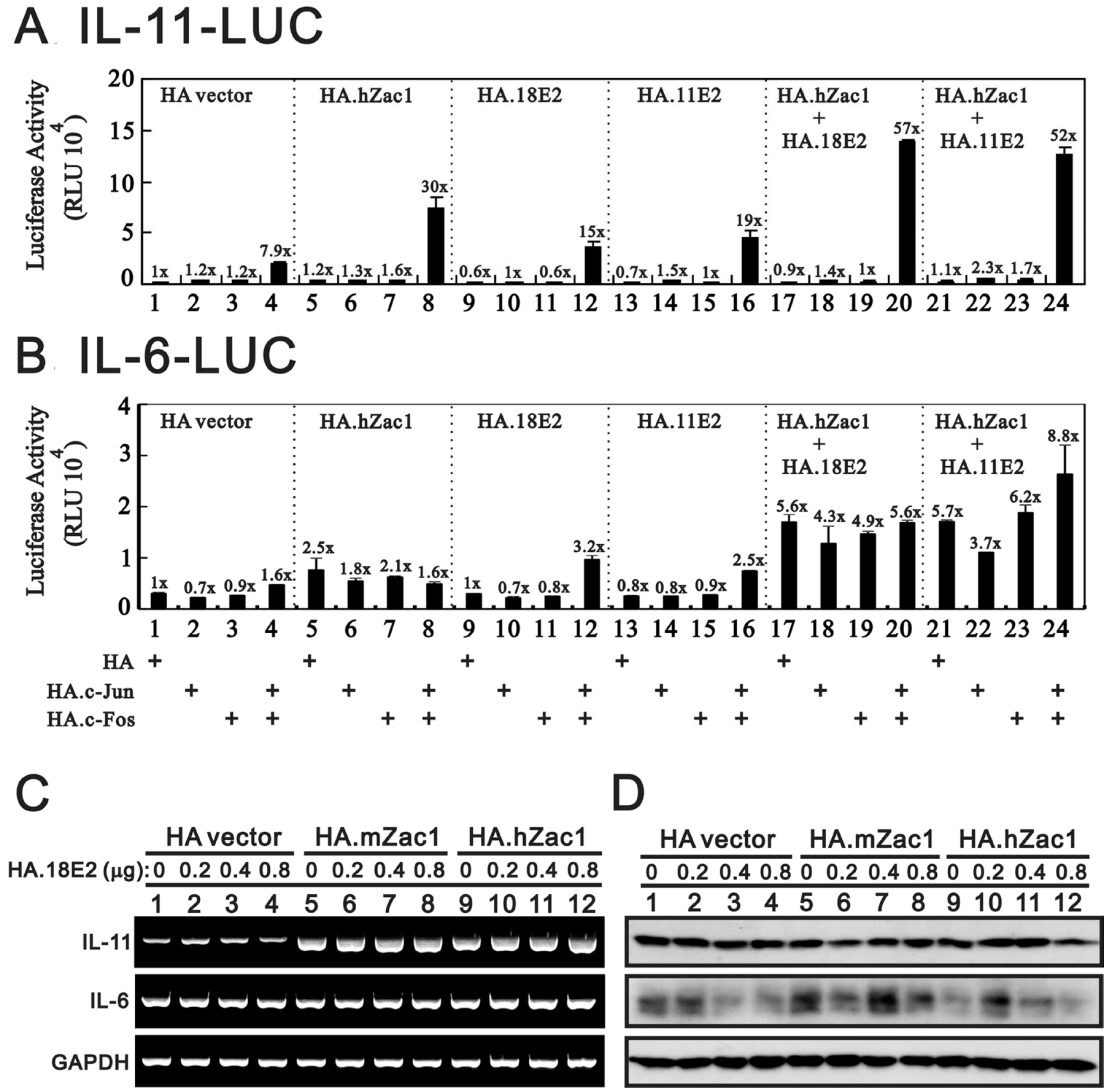
HPV E2 protein within the Zac1/AP1 complex enhances *IL-11* promoter activities HeLa cells were transiently transfected for 36 h with 0.5 μg of *IL-11*-LUC (**A**) or *IL-6*-LUC (**B**) along with 0.5 μg of pSG5.HA, pSG5.HA.c-Jun, pSG5.HA.c-Fos, pSG5.HA.hZac1, pSG5.HA.11E2 or pSG5.HA.18E2. Cells were then harvested for luciferase reporter assays. The numbers above the columns (A and B) indicate the luciferase activity relative to an index of 1 assigned to the reporter with empty vector. HeLa cells were transiently transfected for 36 h with 0.5 μg of pSG5.HA, pSG5.HA.mZac1, pSG5.HA.hZac1, and pSG5.HA.18E2 along with 0.5 μg of pSG5.HA.18E2. The cells were then collected and subjected to RT-PCR analysis of *IL11*, *IL-6*, *and GAPDH* (loading control) mRNA expression (**C**) and immunoblot analysis of IL-11, IL-6, and ACTN (loading control) protein expression (**D**). Results are representative of three independent experiments.

Our previous work showed that Zac1 physically and functionally interacts with AP-1 and HPV E2 proteins [26, 29]. Thus, the effects of c-Jun/c-Fos and PMA on endogenous *IL-11* expression prompted us to examine whether c-Jun/c-Fos might work with Zac1 and HPV E2 proteins in the *IL-11* promoter system. We observed that the c-Jun/c-Fos complex worked synergistically with hZac1, HPV E2, and both in combination in the *IL-11* promoter reporter system (Figure 4A, histograms 1 and 17–24). In general, there was no apparent effect on the *IL-6* promoter reporter system, except for the combination of hZac1 and HPV E2 proteins (Figure 4B, histograms 1, 4, 8, 12, 16, 20, 24). We further examined the effects of the combination of mouse or human Zac1 and HPV18 E2 on *IL-11*, *IL-6*, and *SOCS3* levels using RT-PCR and western blotting. Our data showed that both mouse and human Zac1 acted synergistically with E2 to induce *IL-11* transcription, but not translation, in HeLa cells (Figure 4C, 4D). No apparent effect on the *IL-6* mRNA was observed, but the combination randomly affected IL-6 proteins.

### HIF-1α acted synergistically with AP-1 to enhance *IL-11* promoter activity in HeLa cells

**Figure 5:**
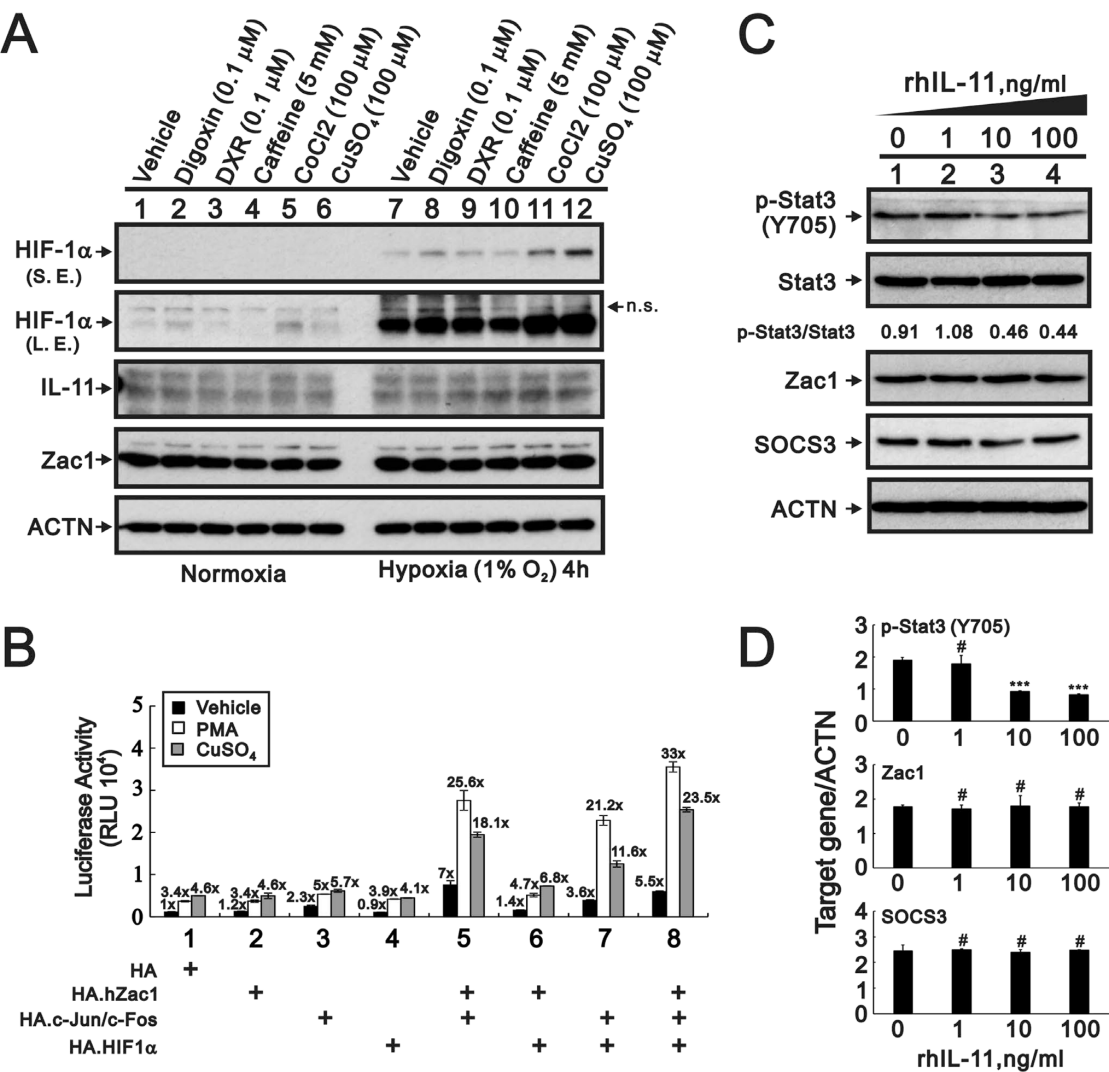
Expression of IL-11 protein is modulated by cobalt chloride, copper sulfate, and hypoxia (**A**) HeLa cells were cultured for 4 h in the presence of digoxin (0.1 µM), DXR (0.1 µM), caffeine (5 mM), CoCl_2_ (100 µM), or CuSO_4_ (100 µM). The cells were then collected and subjected to Western blot analysis for detection of HIF1α, IL-11, Zac1, and PCNA (loading control) expression. S.E.: shorter exposure, L.E.: longer exposure, n.s.: not specific band. (**B**) HeLa cells were transiently transfected with 0.5 μg of *IL-11*-LUC along with 0.5 μg of pSG5.HA, pSG5.HA.HIF-1α, pSG5.HA.c-Jun, or pSG5.HA.c-Fos in the presence of vehicle, PMA, or copper sulfate for 36 h. Cells were then harvested for luciferase reporter assays. The numbers above the columns indicate the luciferase activity relative to an index of 1 assigned to the reporter with empty vector and vehicle. (**C**–**D**) HeLa cells were treated for 24 h with indicated amounts of rIL-11. (C) The cells were collected and subjected to immunoblot analysis for the detection of p-Stat3 (Y705), Stat3, Zac1, SOCS3, and ACTN (loading control) expression. (D) Quantitative analysis of proteins is presented as the mean ± S.D. of at least three independent experiments; ^#^*p* > 0.05 and ^***^p < 0.001 vs the level of ACTN (Student’s *t*-test).

Hypoxia can induce *IL-11* expression in the human colon cancer cell line, HCT116, through cooperative interactions between HIF-1α and AP-1 within the *IL-11* promoter [43]. We treated HeLa cells with the hypoxia mimetic, cobalt chloride (CoCl_2_), or 1% O_2_ for 4 h. Neither CoCl_2_ nor 1% O_2_ induced *IL-11* expression (Figure 5A). The Zac1/AP-1, Zac1/HIF-1α, and HIF-1α/AP-1 complexes acted synergistically within the *IL-11* promoter region in PMA- and copper sulfate-treated HeLa cells (Figure 5B). The Zac1/HIF-1α complex induced the greatest amount of *IL-11* promoter activity.

Multiple studies have shown that the IL-11/IL-11R/STAT3/NF-κB signaling axis plays important roles in tumor growth, angiogenesis, and metastasis [44–52]. We examined the functional role of IL-11 in HeLa cells using a recombinant IL-11 (rIL-11) protein. Our western blotting results showed that p-Stat3 (Y705) and the p-Stat3(Y705)/Stat3 ratio were decreased in a rIL-11 dependent manner in HeLa cells (Figure 5C and 5D). Zacl and SOCS3 were not affected by the addition of rIL-11.

### *ZAC1* silencing downregulated *IL-11* expression

**Figure 6:**
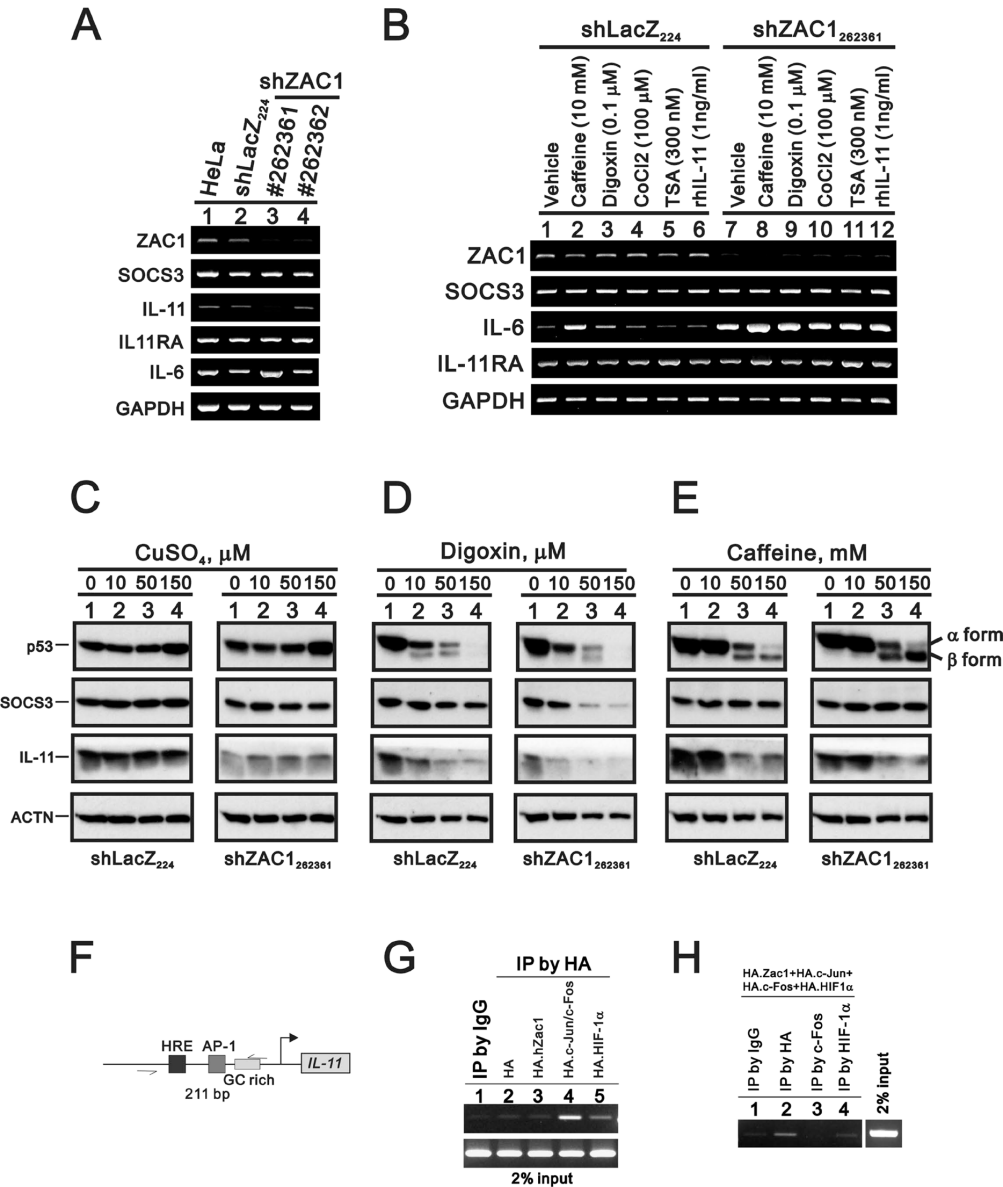
Effects of copper sulfate, digoxin, and caffeine on p53 and SOCS3 expression in *ZAC1* knockdown cells (**A**) Parental HeLa cells, an shLacZ control clone and two shZAC1 knockdown clones were collected and subjected to RT-PCR analysis of *ZAC1*, *SOCS3 IL-11*, *IL-6*, *IL-11Rα*, *and GAPDH* (loading control) mRNA expression. (**B**) HeLa shLacZ and shZAC_1262361_ cells were treated for 20 h with caffeine (10 mM), digoxin (0.1 µM), CoCl_2_ (100 µM), TSA (300 nM), or rIL-11 (1 ng/ml), after which they were subjected to RT-PCR analysis of *ZAC1*, *SOCS3 IL-11*, *IL-6*, *IL-11Rα*, *and GAPDH* (loading control) mRNA expression. HeLa shLacZ and shZAC_1262361_ cells were treated for 20 h with indicated amounts of copper sulfate (**C**), digoxin (**D**), or caffeine (**E**) and subjected to immunoblot analysis for the detection of p53, SOCS3, IL11, and ACTN (loading control) protein expression. (**F**) Schematic representation of the hypoxia responsive element (HRE), AP-1 binding element, and GC-rich region of the *IL-11* promoter. (**G**–**H**) HeLa cells were transiently transfected for 36 h with 4 μg of pSG5HA vector, pSG5HA.Zac1, pSG5HA.c-Jun+pSG5HA.c-Fos, or pSG5HA.HIF-1α (G) or with 2 μg of pSG5HA.Zac1, pSG5HA.c-Jun+pSG5HA.c-Fos, and pSG5HA.HIF-1α (H). Cell lysates were immunoprecipitated using anti-IgG and anti-HA antibodies (G) or with anti-IgG, anti-HA, anti-c-Fos, or anti-HIF-1α antibody (H) and then subjected to PCR analysis. Results are representative of three independent experiments.

We stably silenced *ZAC1* expression in HeLa cells. The silencing efficacy of clone #262361 was better than that of #262362 (Figure 6A). Our data suggested that *IL-11*, not *SOCS3* or *IL-11Rα*, was suppressed in HeLa cells. Caffeine, digoxin, TSA, cobalt chloride, and rIL-11 were employed separately to examine their impacts on ZAC1-induced *IL-11* expression in HeLa cells. We observed no apparent effects on ZAC1 target mRNA expression (Figure 6B). We further examined the effects of copper sulfate, digoxin, and caffeine on p53, SOCS3, and IL-11 proteins in shZAC1 HeLa cells. All three of these agents suppressed IL-11 protein expression in these cells (Figure 6C–6E), including copper sulfate decreased IL-11 expression, digoxin selectively decreased SOCS3 and IL-11 expression, and caffeine decreased IL-11 expression. Consistent with our previous work, digoxin and caffeine increased the β form of p53 in shZAC1 HeLa cells [53, 54].

We further assessed ZAC1-regulated *IL-11* expression using the chromatin immunoprecipitation (ChIP) assay in HeLa cells. Previous studies showed that the *IL-11* promoter region contains two AP-1 sites adjacent to a GC-rich region, a potential Zac1-binding site (Figure 6F). Our ChIP analysis demonstrated that ZAC1 failed to directly bind this 211 bp fragment, but c-Fos did (Figure 6G). However, Zac1 might primarily bind the AP-1 site of the *IL-11* promoter in complex with AP-1 in HeLa cells (Figure 6H).

### The IL-6/IL-11 ratio is clinically relevant for guiding OA treatment options

**Figure 7:**
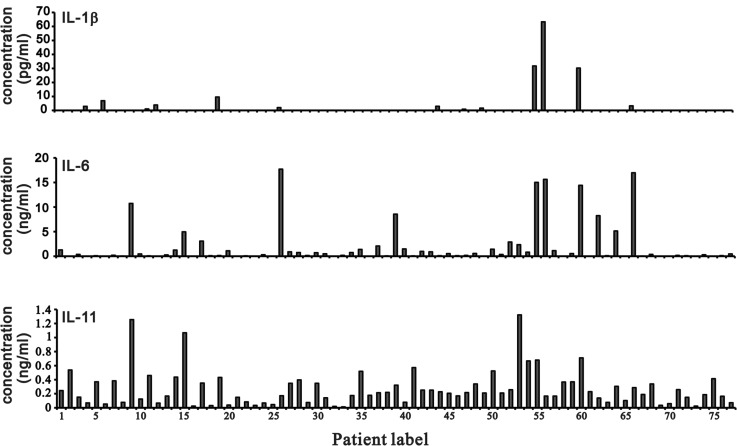
IL-1β, IL-6, and IL-11 levels in synovial fluid from OA patients IL-1β, IL-6, and IL-11 levels were measured in samples of synovial fluid from OA patients using a BD™ CBA Human Soluble Protein Flex Set System.

Cartilage degradation and erosion are important pathogenetic mechanisms in OA [6]. Chemokines and cytokines play key roles in OA pathogenesis. We analyzed the abundance of various cytokines, including IL-1β, IL-6, and IL-11 from the synovial fluids of 77 OA patients using the Cytometric Bead Array (CBA) flex sets (Figure 7). We compared IL-6 and IL-11 median values from the CBA data to define high vs. low IL-6 or IL-11 levels in this study (Table 3). We analyzed 65 OA cases to identify trends in IL-6 level, which was the primary determinant for aggressive vs. conventional clinical treatment (Table 4). In general, high IL-6 level of OA patients received the aggressive treatment, whereas high IL-11 level had the possibility to receive the conventional treatment. Most of low IL-6 level of OA patients received the conventional treatment, whereas low IL-11 level had the possibility to receive the aggressive treatment. Based on the analysis, we further found one OA case with high IL-6 and low IL-11 levels (10731 ng/ml IL-6 vs. 1255 ng/ml IL-11) in the synovial fluid of the right knee associated with recurrent joint effusion and progressive OA joint damage. In contrast, a patient with high IL-11 and low IL-6 levels (538 ng/ml IL-11 vs. 15 ng/ml IL-6) in the left knee responded well to conventional treatment over two years. Thus, the use of high vs. low IL-11 or IL-6 levels to determine OA treatment options requires additional clinical study.

**Table 3:** Summary of IL-6 and IL-11 values of OA patients

		IL-6		IL-11	
		Statistic	Standard error	Statistic	Standard error
Mean		2005.86	504.37	295.49	32.91
95% Confidence interval for mean	Lower bound	998.28		229.75	
	Upper Bound	3013.45		361.24	
**Median**		**371.09**		**219.73**	
Skewness		2.63	.297	2.10	.297
Kurtosis		6.09	.586	5.36	.586

**Table 4:** Summary of aggressive or conventional treatment of OA patients based on the relative amount of IL-6 and IL-11

			Group		Total
			Aggressive treatment	Conventional treatment	
high IL-6	low IL-11	Count	12	0	12
		Expected Count	5.7	6.3	12.0
		% within IL-6_IL-11	100.0%	0.0%	100.0%
		% within Group	**38.7%**	0.0%	**18.5%**
	high IL-11	Count	18	3	21
		Expected Count	10.0	11.0	21.0
		% within IL-6_IL-11	85.7%	14.3%	100.0%
		% within Group	**58.1%**	8.8%	**32.3%**
Low IL-6	low IL-11	Count	1	19	20
		Expected Count	9.5	10.5	20.0
		% within IL-6_IL-11	5.0%	95.0%	100.0%
		% within Group	3.2%	**55.9%**	**30.8%**
	high IL-11	Count	0	12	12
		Expected Count	5.7	6.3	12.0
		% within IL-6_IL-11	0.0%	100.0%	100.0%
		% within Group	0.0%	**35.3%**	**18.5%**
Total		Count	31	34	65
		Expected Count	31.0	34.0	65.0
		% within IL-6_IL-11	**47.7%**	**52.3%**	100.0%
		% within Group	100.0%	100.0%	100.0%

### Synoviocytes and chondrocytes from OA patients

**Figure 8:**
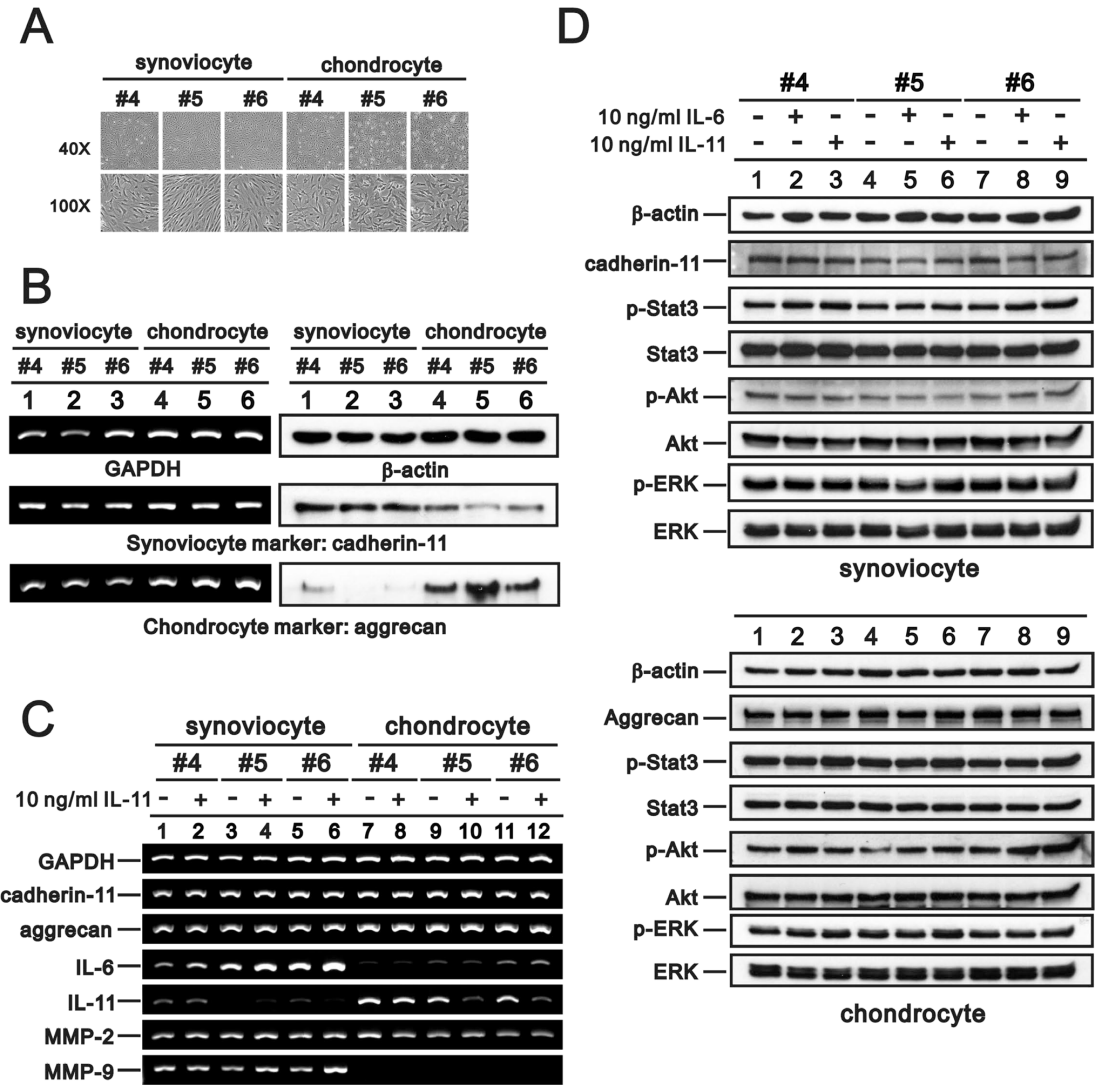
Characteristics of primary synoviocytes and chondrocytes Synoviocytes and chondrocytes from OA patients were observed under a light microscope (**A**) and subjected to RT-PCR analysis of *cadherin-11*, *aggrecan*, *and GAPDH* (loading control) mRNA expression and immunoblot analysis of cadherin-11, aggrecan, and β-actin (loading control) protein expression (**B**). (**C**, **D**) Synoviocytes and chondrocytes were treated with 10 ng/ml rIL-11 for 20 h, after which the cells were subjected to RT-PCR analysis of *cadherin-11*, *aggrecan*, *IL-6*, *IL-11*, *MMP-2*, *MMP-9*, *and GAPDH* (loading control) mRNA expression (C) and immunoblot analysis of cadherin-11 (for synoviocytes), aggrecan (for chondrocytes), p-Stat3, Stat3, p-ERK, ERK, p-Akt, Akt, and β-actin (loading control) protein expression (D). Results are representative of three independent experiments.

To investigate the possible effects of IL-6 and IL-11 on OA progression, we cultured synoviocytes and chondrocytes from OA patient synovial tissue and articular hyaline cartilage, respectively, and measured Il-6 and IL-11 levels. We assessed cell morphology and verified cell characteristics using RT-PCR and western blotting analysis (Figure 8A and 8B; *cadherin-11*: synoviocyte marker, *aggrecan*: chondrocyte marker) [55, 56]. We measured the effects of 10 ng/ml rIL-11 on gene expression in synoviocytes and chondrocytes, including that of *cadherin-11*, *aggrecan*, *IL-6*, *IL-11*, *matrix metalloproteinase-2* (*MMP-2*), and *MMP-9*. IL-11 induced *IL-6* and *MMP-9* expression in synoviocytes and suppressed *IL-11* expression in chondrocytes (Figure 8C). We observed no effects on *cadherin-11*, *aggrecan*, or *MMP-2* levels. IL-11 and IL-6 increased Akt phosphorylation, but did not impact other signaling pathways, including STAT3, m-TOR, and ERK (Figure 8D).

## DISCUSSION

**Figure 9:**
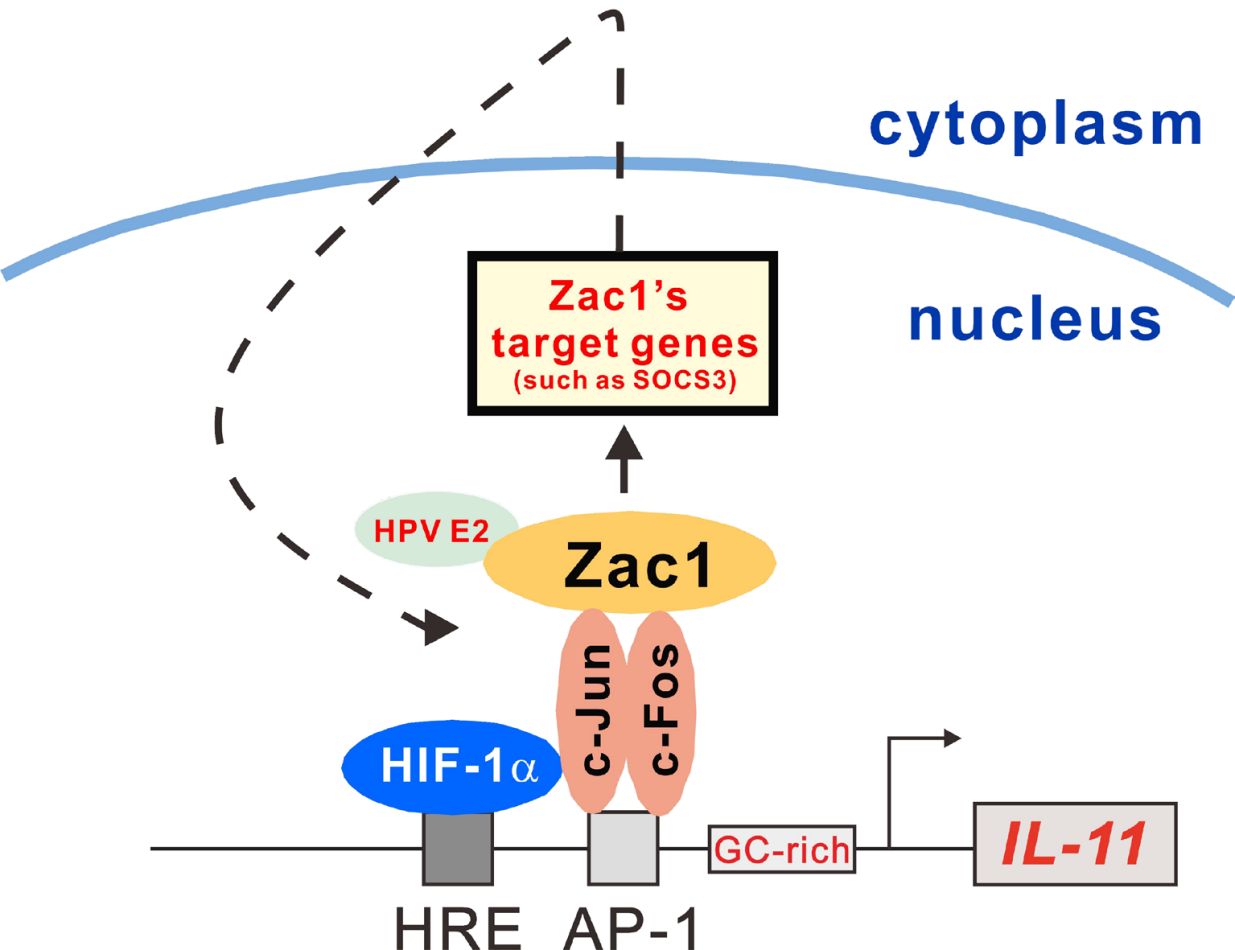
Schematic representation of the HRE and AP-1 binding element and the GC-rich region of the *IL-11* promoter Transcription factor Zac1 may not only induce transcription of target genes, such as SOCS3, it may also bind to host cellular proteins involved into the *IL-11* gene regulation, such as HPV E2. Zac1 might also act as a transcriptional factor coactivator for AP-1 and HIF-1α to form induction complexes on the *IL-11* promoter in HeLa cells.

In this work, we verified that Zac1 induced *IL-11* expression in HeLa cells. Zac1 might indirectly bind the IL-11 promoter as a transcription factor or/and cofactor complexed with AP-1, HIF-1α, or other transcription factors (Figure 9). Zac1 may also induce IL-6 expression under specific conditions. Finally, we observed that high or low IL6 or IL-11 levels might serve as indicators to determine which treatment strategy would best benefit a given OA patient. Our work provides novel insight into IL-11 expression regulation and the clinical relevance of the IL-11/IL-6 ratio in OA patients.

A functional IL-11Rα subunit might trigger signal transduction by IL-11 in human cancer cells cultured under hypoxic conditions [43]. Zac1 also induced IL-11Rα, and recombinant IL-11 proteins failed to activate STAT3 phosphorylation in this study. Most of current experimental conditions might not be performed in the hypoxic situation and the expression of *IL-11* mRNA is inconsistent with a hypoxia-inducible gene in HeLa cells. One study showed that *IL-11* and *IL-11Rα* are co-expressed in rheumatoid arthritis synovitis fluid fibroblasts and endothelial cells and thus interconnect the function of these two cell types, whereas macrophages are not IL-11 responder cells because of no IL-11Rα expression [57]. Therefore, activation of the IL-11/IL-11Rα complex under hypoxic conditions must be addressed with respect to tumorigenesis. Previous studies demonstrated that copper sulfate induced HIF-1α expression via the c-Fos and hypoxia reduced some AP-1 protein expression [58, 59]. However, our results suggested that copper sulfate might positively or negatively regulate HIF-1α expression depending on hypoxic conditions.

Cartilage consists of two main extracellular matrix (ECM) macromolecules: type II collagen (COL2A1) and a large aggregating proteoglycan, aggrecan [10]. COL2A1, the major cartilage collagen, and types IX and X encoding so-called cartilage-specific collagens are the predominant ECM structural proteins involved into OA pathogenesis. COL9A3 is associated with knee OA in the Japanese population [60] and was among the top five Zac1-induced genes in our study. Zac1 expression patterns correlated with COL2A1 distribution during cartilage development [61]. Hence, ZAC1 might promote OA progression through regulation of cytokines, such as IL-11, or the collagens, such as COL2A1 or COL9A3, although verification of this hypothesis will require further study.

IL-6 and its family of proteins, including oncostatin M, are among the most prominently elevated cytokines in the OA inflammatory response [62]. Many studies have correlated of IL-6 levels in synovial fluid with cartilage pathology and associated outcomes in knee OA. Unlike IL-6, IL-11 is well studied with respect to OA [7–10]. MMP-9 activity is crucial for the destruction of articular cartilage in OA. Our data suggest that ZAC1 upregulates both IL-6 and IL-11 via AP-1 family proteins. Zac1 may directly induce expression of *MMP*-related genes through AP-1- and Sp1- dependent pathways. Thus, we propose that high vs. low IL-6 or IL-11 levels can be indicators for OA clinical treatments. However, there are likely additional factors within synovial fluid that could impact treatment decisions, and these require further study.

## MATERIALS AND METHODS

### Tetracycline-induced *Zac1* and short-hairpin *Zac1* RNA in HeLa cells

We established tetracycline-induced *Zac1* expression in HeLa cells using the standard protocols for Tet-On Inducible Gene Expression System (Clontch, USA). Two vectors, pTet-On and pTRE-Zac1 (or control vector pTRE-LacZ), were screened and selected using G418 and hygromycin, respectively (Clontch, USA). Positive clones were expanded and sorted for Zac1 expression induced by doxycycline.

Zac1shRNA-containing lentiviral vectors were purchased from the National RNAi Core Facility (Academia Sinica, Taiwan, Republic of China) and prepared using standard protocols. Cells were infected with the indicated lentiviruses in selection medium containing 2 μg/ml polybrene. Forty-eight hours after infection, the cells were treated with 8 μg/ml puromycin for selection of resistant clones.

### Cell culture, plasmid DNAs, and chemicals

HeLa cells were cultured in Dulbecco’s modified Eagle’s medium (DMEM) supplemented with 10% fetal bovine serum (FBS; Invitrogen, CA, USA) and 1% penicillin-streptomycin (Invitrogen). The pSG5.HA.Zac1, AP-1, and HPV E2 expression plasmids and *IL-6 (*−*1260/+84)*-LUC reporter have been described previously [26, 28, 29, 63]. *IL-11(-750/+70)*-LUC and *SOCS3* promoter fragments were introduced into the pGL3-LUC vector at the BamHI/XhoI site. An expression vector for HIF-1α was constructed from a PCR fragment and introduced into the pSG5.HA vector at the EcoRI/XhoI site. Recombinant IL-6 and IL-11 proteins were purchased from R&D Systems (MN, USA). PMA, TSA, caffeine, digoxin, cobalt chloride, and copper sulfate were purchased from Sigma-Aldrich (MO, USA).

### Total RNA isolation, mRNAs expression profiles, and first strand cDNA synthesis

Total RNA was isolated from HeLa cells using TRIzol reagent (Invitrogen) following the instructions provided by the manufacturer. Gene expression profiles were determined using the HOA OneArray method and HmiOA v5. RNA quantitation was provided by the Phalanx Biotech Group (HsinChu, Taiwan, ROC). The pass criteria indicating acceptable RNA purity using NanoDrop ND-1000 were the following absorbance ratios A260/A280 ≥ 1.8 and A260/A230 ≥ 1.5. RNA integrity (RIN) values were determined using an Agilent RNA 6000 Nano assay. The pass criterion was a value ≥ 6. Contamination by genomic DNA was evaluated using gel electrophoresis. Data were processed though the Rosetta Resolver^®^ System (Rosetta Biosoftware). Reverse transcription for first strand cDNA synthesis was carried out using a ThermoScript^™^ RT-PCR system (Invitrogen) with 1 μg of total RNA. GAPDH was used as an internal control for normalization.

### Luciferase reporter assay

HeLa cells were plated in 24-well plates and transfected using jetPEI (Polyplus Transfection Inc., New York, NY, USA) following the manufacturer’s protocol (Promega luciferase assay kit and DLR2 model). The total DNA was adjusted to 1.0 μg using empty vector. Cells were harvested for luciferase reporter assays using a Promega Luciferase Assay Kit. Values are expressed numerically as relative light units. Luciferase activity is presented as the mean ± SD of three transfected wells and is representative of at least three independent experiments.

### Reverse transcription-polymerase chain reaction (RT-PCR)

One microgram of total RNA was subjected to the reverse transcription using MMLV reverse transcriptase for 60 min at 37° C (Epicentre Biotechnologies, USA). The PCR reactions were run in a Veriti Thermal Cycler (Applied Biosystems, MA, USA). PCR was performed in the linear range (30 cycles) with primers specific for *hZac1*, *IL-11*, *IL-6*, *SOCS3*, *IL-11Rα*, *cadherin-11*, *aggrecan*, *MMP-2*, *MMP-9*, and *GAPDH*. The sequences of the primers for the amplification of target genes are shown in Table 5. The thermocycling conditions were as follows: one cycle at 95° C for 5 mins followed by 30 cycles of 95° C for 45 s, 55° C for 30 s, and 72° C for 40 s. Amplified products were subjected to 1.2 % agarose gel electrophoresis and visualized by staining with ethidium bromide.

**Table 5:** PCR primers used in this study

Gene name	Primer sequence (5′→3')
*hZac1*	Forward: 5'-ttcctcaccctggagaag-3' Reverse: 5'-tccttgcatcctgtgtgg-3'
*IL-11*	Forward: 5'-atgaactgtgtttgccgcctggtc-3' Reverse: 5'-tcacagccgagtcttcagcagcag-3'
*IL-6*	Forward: 5'-atgaactccttctccacaagcgc-3' Reverse: 5'-ctacatttgccgaagagccctca-3'
*SOCS3*	Forward: 5'-atggtcacccacagcaagtttccc-3' Reverse: 5'-ttaaagcggggcatcgtactggtc-3'
*IL-11Rα*	Forward: 5′-acctacatctgccagaccct-3' Reverse: 5′-tggtactgactctacccgca-3'
*cadherin-11*	Forward: 5′-cgtggagggttcagtcggcaga-3′ Reverse: 5′-tactgatactcaggtttgat-3′
*aggrecan*	Forward: 5′-cagaatctagcagtgagacgtc-3' Reverse: 5′-gtctgcagcagttgattctgat-3'
*MMP-2*	Forward: 5′-cctgagatctgcaaacaggacatt-3' Reverse: 5′-ttcttcttcacctcattgtatctcc-3'
*MMP-9*	Forward: 5′-aagtggcaccaccacaacat-3' Reverse: 5′-tttcccatcagcattgccgt-3'
*GAPDH*	Forward: 5'-aacggatttggccgtattggg-3' Reverse: 5'-gggatgaccttgcccacagcc-3'

### Western blotting analysis

For Western blotting, HeLa cells were lysed in RIPA buffer (100 mM Tris-HCl pH 8.0, 150 mM NaCl, 0.1% SDS, and 1% Triton 100) at 4° C, separated by SDS-PAGE, and analyzed by immunoblotting with antibodies against Zac1 (MW: 51 kDa; 1:2000 dilution) and p-Stat 3 (Y705) (MW: 88 kDa; 1:1000 dilution) (Abcam, Cambridge, UK); ACTN (MW: 100 kDa; 1:10000 dilution), aggrecan (MW: 200 kDa; 1:1000 dilution), cadherin-11 (MW: 110 kDa; 1:500 dilution), HIF-1α (MW: 132 kDa; 1:1000 dilution), IL-11 (MW: 23 kDa; 1:1000 dilution), IL-6 (MW: 21/27 kDa; 1:1000 dilution), SOCS3 (MW: 30 kDa; 1:1000 dilution), p53 (MW: 53 kDa; 1:2000 dilution), PCNA (MW: 36 kDa; 1:10000 dilution), and β-actin (MW: 43 kDa; 1:10000 dilution) (Santa Cruz, TX, USA); and Akt (MW: 60 kDa; 1:5000 dilution), p-Akt (MW: 60 kDa; 1:1000 dilution), ERK (MW: 42/44 kDa; 1:2000 dilution), p-ERK (MW: 42/44 kDa; 1:2000 dilution), Stat 3 (MW: 79/86 kDa; 1:1000 dilution), ChIP-grade Hemagglutinin (HA), ChIP-grade c-Fos, and ChIP-grade HIF-1α (Cell Signaling, MA, USA).

### Chromatin Immunoprecipitation (ChIP) assays

ChIP assays were performed according to the manufacturer’s protocol (Cell Signaling Technology, #9003S). Briefly, chromatin/DNA proteins complexes were prepared from HeLa cells transfected for 24 h with 1 μg of pSG5-HA (empty vector), HA-mZac1, HA-Jun or/and HA-HIF-1α. Chemical cross-linking of DNA-proteins was carried out using 1% formaldehyde for 10 min at room temperature. The Cross-linking was quenched by addition of glycine for 5 min at room temperature and followed by two washes with ice-cold PBS. Cells were then scraped into PBS containing Protease Inhibitor Cocktail (PIC). The resultant cell suspension was centrifuged, and the pellet was mixed by inverting the tube every 3 min in buffer A + DTT+ PIC and then incubated for 10 min on ice. The pellet (nuclei) was resuspended in 100 µl of Buffer B +DTT + 0.5 μl of micrococcal nuclease and incubated for 20 min at 37° C with frequent mixing to digest the DNA to lengths of approximately 150–900 bp. The nuclei were then completely lysed by sonication using Q125 sonicator (Qsonica, NY, USA). The lysate was incubated overnight at 4° C with rotation with appropriate ChIP-grade anti-HA, anti-c-Fos, or anti-HIF-1α antibody (Cell Signaling Technology) to immunoprecipitate chromatin. This was followed by incubation 2 h at 4° C with rotation with ChIP-grade protein G magnetic beads. The magnetic beads were washed using buffers supplied with the kit. The eluted DNA was purified using ChIP DNA Clean & Concentrator Kits (Zymo Research, CA, USA) and analyzed using PCR to assess binding of c-Jun, HIF-1α, or Zac1 to the *IL-11* promoter. The PCR primers were designed using criteria described in the kit. A region of the human *IL-11* promoter bearing putative binding sites for both AP-1 and HRE and a GC-rich potential Zac1 binding site were amplified using the following primers: 5′-AGC CTG AGT GTC TGC TCC G-3′ (forward) and 5′-TGA CAC ATC CTG ACT CAC CCT CC-3′ (reverse). The control primers for human *RPL30* exon 3 were provided with the kit. End-point PCR amplification was performed using EmeraldAmp MAX HS PCR Master Mix (Clontech, CA, USA) and using the Applied Biosystems Veriti Mastercycler.

### Patient recruitment and collection of synovial fluid

Synovial fluids were collected from patients who underwent medical examinations at the National Defense Medical Center (Taipei, Taiwan, ROC). The Institutional Review Board of Tri-Service General Hospital, Taipei, Taiwan approved the study protocol, and signed informed consent was obtained from all subjects before study participation (TSGHIRB No:1-104-05-066). Radiographs were reviewed to determine the size and stage of progression of the OA lesions. Synovial fluid was collected from OA patients using a 22-gauge needle and then stored at −80° C until assayed.

### IL-1β, IL-6, and IL-11 measurement

IL-1β, IL-6, and IL-11 levels were measured using a BD™ CBA Human Soluble Protein Flex Set System (Becton Dickson, San Jose, CA) according to the manufacturer’s instructions. For CBA analysis, diluted synovial fluid or standards were mixed with Capture Beads, vortexed, centrifuged, and transferred to tubes containing PE Detection Reagents for incubation and analysis. The complexes were then analyzed using flow cytometry (FACScan argon laser cytometer) to identify particles with fluorescence characteristics of both the bead and the detector. Data were analyzed using FCAP Array software (No. 652099).

### Cultures of chondrocytes and synoviocytes

To culture chondrocytes, samples of articular hyaline cartilage were collected from patients who underwent total knee replacement to treat OA. The cartilage was minced into 1–5 mm^3^ pieces and digested in 5–8 ml of 0.2% collagenase II (Sigma-Aldrich) for 12–16 h at 37° C under 5% CO_2_. The dissociated chondrocytes were then centrifuged, after which the pellets were re-suspended in 5 ml of DMEM/F12 (Invitrogen) containing 15% FBS and 1% penicillin/streptomycin solution, transferred to a culture flask, and incubated for 24 h at 37° C under 5% CO_2_. After refreshing the growth medium, the remaining adherent cells were cultured for an additional 2 weeks, changing the growth medium every 3 days. Chondrocyte passages 1 and 2 were used for experiments.

To culture synoviocytes, samples of synovial tissues were treated with trypsin (10% v/v) for 15 min in a 37° C water bath, and the resultant tissue fragments were transferred to DMEM containing 5% FCS, penicillin-streptomycin-Fungizone and 2 mg/ml clostridial collagenase type IV (Sigma-Aldrich). The fragments were then digested on a gyratory shaker for approximately 3 h to dissociate the synoviocytes. The cells were washed and cultured in T175 flasks for 24 h. After refreshing the medium, the adherent synovial cells were further cultured until experiments were performed.

### Light microscopic analysis of cell morphology

Human chondrocytes (or synoviocytes) grown in 24-well plates to 80% confluence were examined using an Zeiss Axiovert 40 C light microscope (Göttingen, Germany).

### Statistical analysis

Student’s *t*-test was used to compare protein amounts derived from Western blot analysis. The Mann–Whitney *U* test was used to compare levels of IL-11 and IL-6 in samples from recruited OA patients. All values are expressed as means ± SEM. Values of *P* < 0.05 were considered significant.

## Abbreviations

IL: Interleukin
OA: osteoarthritis
Zac1: a zinc finger protein which regulates apoptosis and cell cycle arrest
gp130: glycoprotein 130
*JAK*: Janus tyrosine Kinase
*STAT*: Signal Transducer and Activator of Transcription
PACAP_1_-R: type I pituitary adenylate cyclase-activating polypeptide receptor gene
HPV: human papillomavirus
NRs: nuclear receptors
IL-11Rα: receptor alpha of IL-11
RT-PCR: reverse transcription-polymerase chain reaction
Dox: doxycycline
PMA: phorbol 12-myristate 13-acetate
TSA: trichostatin A
rIL-11: recombinant IL-11
MMP: matrix metalloproteinase
ACTN: α-actinin
HA: hemagglutinin
ChIP: chromatin immunoprecipitation
FACS: fluorescence-activated cell sorting
PI: propidium iodide
